# Supplementary figures and images for: Diabetes mellitus and its association with central obesity, and overweight/obesity among adults in Ethiopia. A systematic review and meta-analysis
Source: PLoS One. 2022 Jun 10;17(6):e0269877. doi: 10.1371/journal.pone.0269877 (PMC9187119; doi:10.1371/journal.pone.0269877)

Table B. Literature screening Microsoft-excel sheet


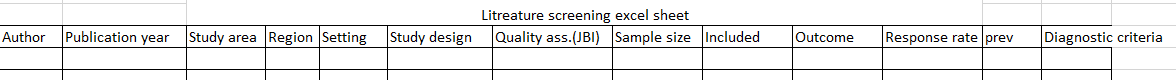

Supplement: S2 Table — (DOCX) [file pone.0269877.s002.docx]

Table C. Quality assessment checklist


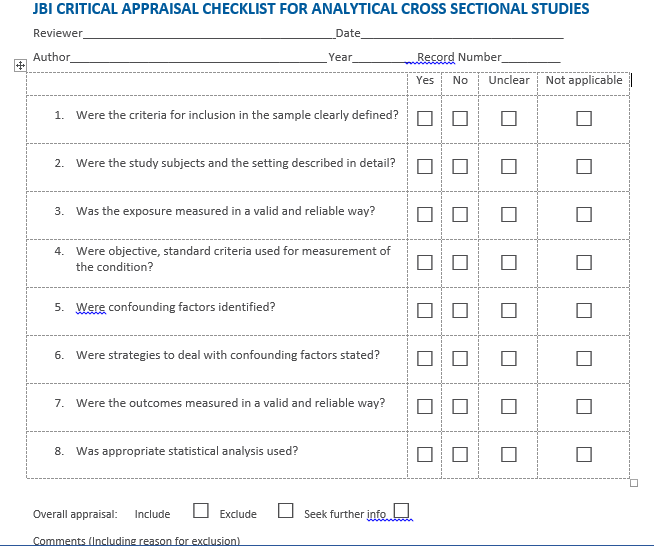

Supplement: S3 Table — (DOCX) [file pone.0269877.s003.docx]

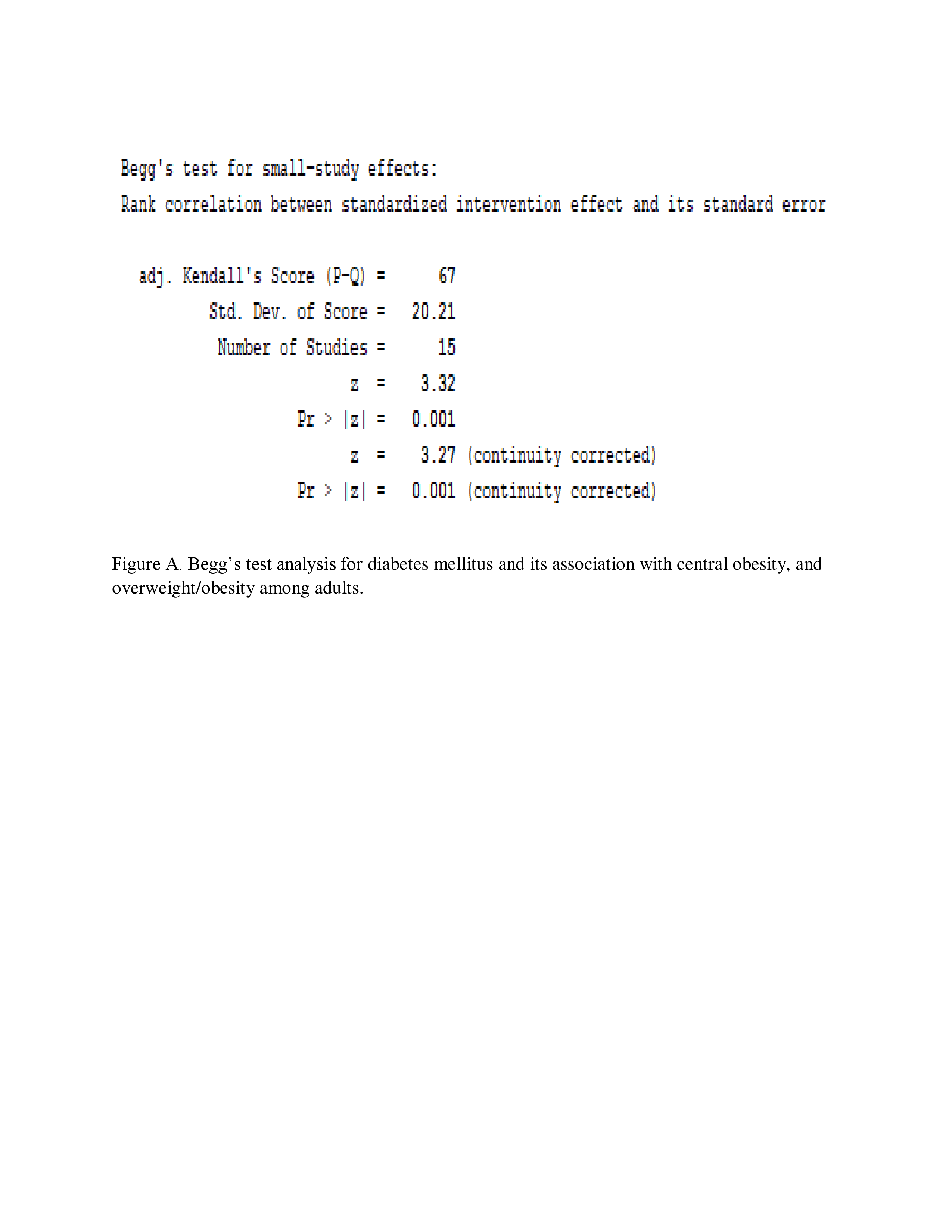

Supplement: S1 Fig — (TIFF) [file pone.0269877.s004.tiff]

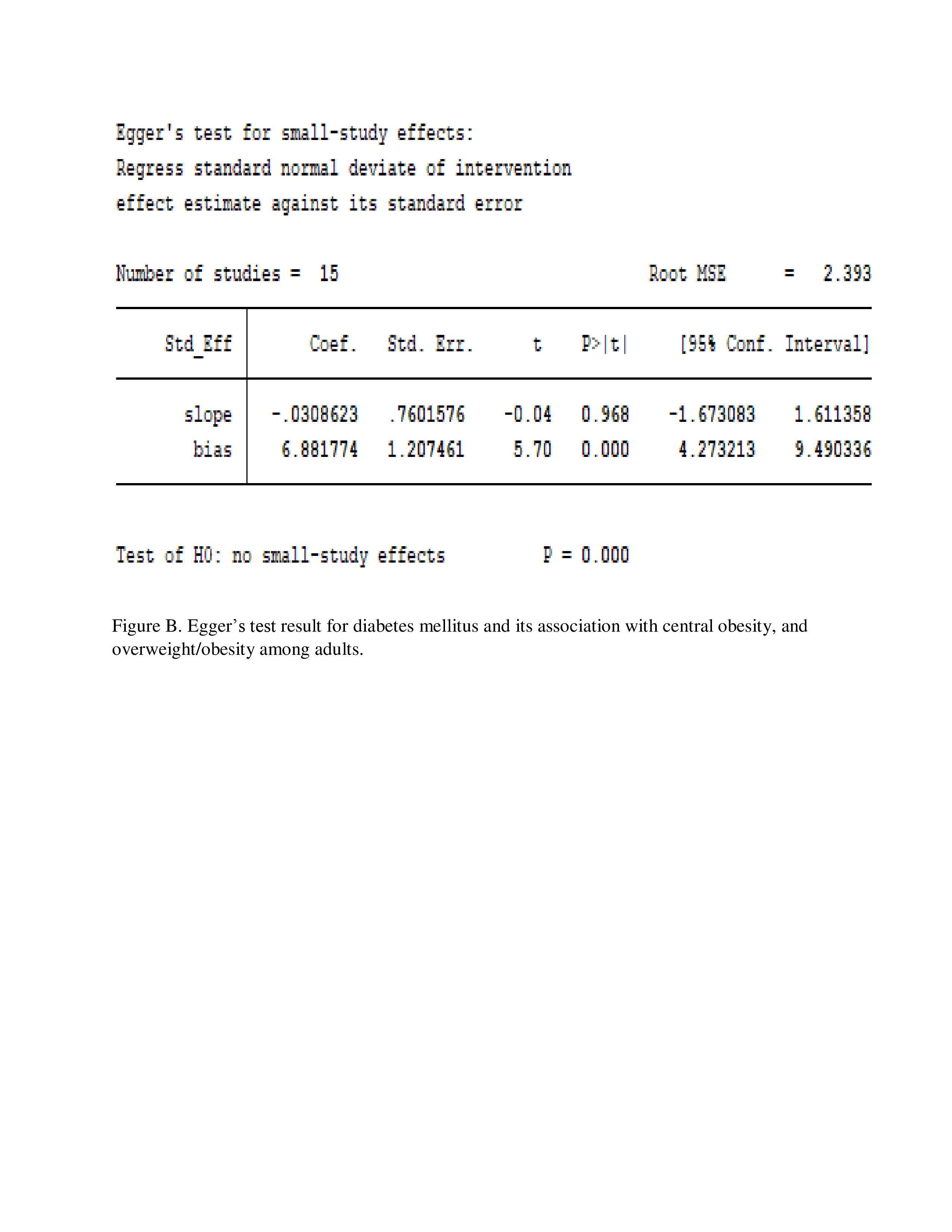

Supplement: S2 Fig — (TIFF) [file pone.0269877.s005.tiff]
