# Supplementary material for: Diabetes mellitus and its association with central obesity, and overweight/obesity among adults in Ethiopia. A systematic review and meta-analysis
Source: PLoS One. 2022 Jun 10;17(6):e0269877. doi: 10.1371/journal.pone.0269877 (PMC9187119; doi:10.1371/journal.pone.0269877)
Supplement: Attachment — Submitted filename: Response_To_Reviewers.docx [file pone.0269877.s006.docx]

|  | Reviewer #1 |  |  |
| --- | --- | --- | --- |
| S/No | Reviewers comment | Authors response | Reference |
| 1 | Line 125: The authors referred to an obsolete version of PRISMA guidelines (Ref. 8). They should revise the analysis following the updated PRISMA guidelines (that have been published early last year). | Thank you very much for your suggestion. It has been  corrected based on the recent PRISMA-2020 guidelines” | page 13 |
| 2 | Line 152: The authors should also perform Begg test | Thank you very much for your insightful comments. As per the reviewer’s comment it has been corrected. | Page 9 |
| 3 | Line 152-153: The authors should specify how sensitivity analysis was performed | Thanks! The authors addressed the given comments. | Page 7 |
| 4 | Table 1: Information regarding the prevalence by sex should be included in the table. | Thanks for your insightful comments. It would have been better if we could have this variable, but most of the eligible studies lack this variable. So, we are unable to assess the prevalence based on sex. | ---- |
| 5 | The authors should consider to refer to doi: 10.1016/j.numecd.2020.03.005 in their manuscript | Thanks for the comments. We found this literature crucial for our review. | Page 11 and reference number 30. |
| 6 | The limitations of the analysis should be more deeply discussed | As per the comment it has be discussed | Page 12 |
| 7 | English language needs to be revised | Thanks!. We authors made a correction as per the given comment. | Throughout the document |
